# Sound mind, irrational behavior?

**DOI:** 10.3389/fpsyg.2014.00433

**Published:** 2014-05-16

**Authors:** R. Kim Guenther, Matthew H. Olson

**Affiliations:** Department of Psychology, Hamline UniversitySaint Paul, MN, USA

**Keywords:** reasoning, decision making, rationality, illusion of control, folk psychology, irrational thinking

Jonathan St. B. T. Evans, in his engaging article “Rationality and the illusion of choice” (Evans, 2014) argues that erroneous answers to questions posed in the study of reasoning and decision-making (RDM) ought not be viewed as *irrational*, just as errors in other cognitive domains are not viewed as irrational. He attributes misuse of the term partly to use of normative models borrowed from economics or mathematics but even more to an RDM assumption of conscious, controlled rationality. Our intent is to respond to the main tenets of Evans's argument and suggest, instead, that attributing RDM errors to irrationality is proper.

Evans (2014); Evans (paragraph 12) writes: “How can we both presuppose rationality and then infer irrationality from errors? Researchers in no other fields of cognitive psychology do this, inferring instead cognitive limitations from errors.” Thus, perceptual inaccuracy is described using language of perceptual illusion; suboptimal memory performance is forgetting or distortion. In experimental tasks common to RDM, however, the term “rational” pertains specifically to normative prescriptions for optimal performance. Performance that systematically violates those prescriptions, even when outcomes are consistently substandard or even negative, is labeled “irrational.” See, for example, Ariely (2008).

Further, Evans (2014, paragraph 5) says “In most of cognitive psychology there is little or no debate about what constitutes an error. A signal is present or not and hence detected or not by the participants' judgment…” Evans' language is unmistakably from Signal Detection Theory (SDT) (Tanner and Swets, 1954), and it is instructive to explore SDT further. In SDT, errors are not random mistakes or lapses of attention. They are, instead, the systematic result of a problem-solving strategy (based on a formal normative model) that cannot yield error-free performance. Although not optimized as in SDT, many errors in RDM occur because people who are paying attention, motivated, educated, and not otherwise cognitively compromised, violate normative prescriptions for behavior in systematic ways (e.g., the framing effect or neglect of base rate). Using standard dictionary definitions, the word “irrational” fits many error-types in the RDM domain, but perhaps not in other cognitive domains. Forgetting, for example, is not a lack of reasoning, logic, or sound judgment; but then again, there are no formal, normative rules for remembering. Much of the research in RDM, however, observes behavior when optimal normative prescriptions are available but are either ignored or misused by participants.

To augment his thesis, Evans refers to his theory of old mind/new mind, whose theoretical drivers (instrumental learning, hypothetical thinking, and their conflicts) are not themselves characterized as rational or irrational. The implication seems to be that this theory, and similar theories (Stanovich, 2011), eliminates the need for using the term “irrationality.” But there is no necessity that a theory's drivers resemble predicted performance errors, just as a theory of memory need not have as a theoretical driver a mechanism that forgets. Additionally, it is the new mind component of hypothetical thinking, uniquely human in Evan's view, to which normative tenets of reasoning and logic apply, thereby allaying Evan's concern that only human animals are characterized using the rational/irrational distinction.

Not all errors in the RDM domain are properly labeled irrational. Often they result from cognitive capacity limitations, such as a limited working memory, attentional focus, and stimulus-response competition. Capacity limitations are likely responsible for foiling aspects of syllogistic reasoning, as one example, and sometimes lead to using heuristics that may work at least satisfactorily, as when people guess that familiar cities have large populations (Goldstein and Gigerenzer, 2002). Still, even when optimal strategies are available and not cognitively burdensome, people adhere to suboptimal strategies that consistently produce systematic errors, as observed, for example, in the framing effect and the gambling fallacy.

Although not quite made explicit, Evans's central thesis is that characterizing suboptimal RDM performance as “irrational” is specifically *pejorative*, in a way that such characterizations in other cognitive domains are not. With respect to errors in other cognitive tasks, he writes (Evans, 2014, paragraph 3): “In no case is the *person'* held responsible and denounced as irrational.” It is not insulting to label people as forgetful, but it seems unkind to label them as irrational. Evans claims that the origin of the pejorative “irrational” label is the widespread but mistaken belief that the conscious self is in charge of our actions, and that a belief in this sort of “free will” (our summary phrase for Evan's description) is invoked in the RDM domain, especially decision-making, but not in domains like memory or perception. Inconveniently for his claim, we find no evidence that researchers in RDM make use of or imply such free will in their theoretical explanations. Moreover, the strategic basis of thinking that is labeled irrational is typically understood to be outside of conscious awareness.

If “irrational” is pejorative, then RDM researchers ought to find another name for it, as in the case of people with cognitive deficits now being labeled “cognitively delayed,” but who used to be labeled “feeble-minded.” Unfortunately for the pejorative argument, it is unclear that the label “irrational” is similarly insulting, given its use in the context of characterizing *systematic performance* that may suffice but not optimize, and clearly does not divide people into categories in which some are rational and others are irrational. In the domain of RDM, it is the strategy, not the person, that is irrational, but to fault the strategy is not to “denounce” the person.

## Conflict of interest statement

The authors declare that the research was conducted in the absence of any commercial or financial relationships that could be construed as a potential conflict of interest.
